# Muscle Nutritive Metabolism Changes after Dietary Fishmeal Replaced by Cottonseed Meal in Golden Pompano (*Trachinotus ovatus*)

**DOI:** 10.3390/metabo12070576

**Published:** 2022-06-22

**Authors:** Yawen Qin, Chaoqun He, Haoyu Geng, Wenqiang Wang, Peng Yang, Kangsen Mai, Fei Song

**Affiliations:** 1 Guangzhou Key Laboratory of Subtropical Biodiversity and Biomonitoring, Guangdong Provincial Key Laboratory for Healthy and Safe Aquaculture, Institute of Modern Aquaculture Science and Engineering (IMASE), College of Life Science, South China Normal University, Guangzhou 510631, China; 2020022760@m.scnu.edu.cn (Y.Q.); 2020022843@m.scnu.edu.cn (C.H.); 2021023073@m.scnu.edu.cn (H.G.); 2019022514@m.scnu.edu.cn (W.W.); 2019022432@m.scnu.edu.cn (P.Y.); kmai@ouc.edu.cn (K.M.); 2 Southern Marine Science and Engineering Guangdong Laboratory (Zhuhai), Zhuhai 519000, China; 3 Southern Marine Science and Engineering Guangdong Laboratory, Zhanjiang 524025, China

**Keywords:** fish nutrition, marine aquaculture, replacement, cottonseed meal, physiobiochemical, nutrient

## Abstract

Our previous study demonstrated that based on growth performance and feed utilization, cottonseed meal (CSM) could substitute 20% fishmeal (FM) without adverse effect on golden pompano (*Trachinotus ovatus*). Muscle deposition was also an important indicator to evaluate the efficiency of alternative protein sources. Therefore, the present study was conducted to explore the changes of physiobiochemical and nutrient metabolism in muscle after FM replaced by CSM. Four isonitrogenous and isolipidic experimental diets (42.5% crude protein, 14.0% crude lipid) were formulated to replace 0% (CSM0 diet), 20% (CSM20 diet), 40% (CSM40 diet), and 60% (CSM60 diet) of FM with CSM. Juvenile fish (24.8 ± 0.02 g) were fed each diet for 6 weeks. The results presented, which, compared with the CSM0 diet, CSM20 and CSM40 diets, had no effect on changing the muscle proximate composition and free essential amino acid (EAA) concentration. For glycolipid metabolism, the CSM20 diet did not change the mRNA expression of hexokinase (hk), glucose transport protein 4 (glut4), glucagon-like peptide 1 receptor (glp-1r), while over 20% replacement impaired glucose metabolism. However, CSM20 and CSM40 diets had no effect on altering lipid metabolism. Mechanistically, compared with the CSM0 diet, the CSM20 diet did not change muscle nutritive metabolism through keeping the activities of the nutrient sensing signaling pathways stable. Higher replacement would break this balance and lead to muscle nutritive metabolism disorders. Based on the results, CSM could substitute 20–40% FM without affecting the muscle nutritive deposition. All data supplemented the powerful support for our previous conclusion that CSM could successfully replace 20% FM based on growth performance.

## 1. Introduction

Muscle is the largest tissue of teleost fish, accounting for more than 60% of the body weight. It provides premium proteins to meet the requirement for humans [1,2]. Previous studies demonstrated that alternative proteins replace fishmeal (FM) disturbed muscle nutrient metabolism and further resulted in the muscle growth restriction considered as an important limiting factor affecting substitution efficiency [3,4]. Additionally, the main goal of the aquaculture was to improve feed utilization, which in turn efficiently promoted muscle deposition [5,6,7]. Therefore, muscle nutrient deposition was the important indicator to assess the efficiency of alternative protein sources in substituting FM.

As in mammals, fish muscle growth is dependent on the recruitment of new muscle fiber (hyperplasia) and growth of existing fiber (hypertrophy) [8,9,10]. The essence of hypertrophy is the deposition of nutrients in muscle [11,12]. These processes are controlled by nutrient sensing signaling pathways at both cellular and systemic levels [13]. Amino acid (AA) transporters are regarded as the first sensor and carrier facilitate AA across the cell membrane, resulting in changes of free AA concentrations in plasma and tissues [14,15,16]. The target of rapamycin (TOR) and amino acid response (AAR) pathways could sense the nutrient status, especially AA concentrations, at the cellular level [17,18,19]. Moreover, the insulin/insulin-like growth factor (IGF) system is the main nutrient sensing pathway at a systemic level [20]. As the upstream, IGF through phosphoinositide-3-kinase (PI3K)/protein kinase B (AKT) regulated TOR and AAR signaling to modify nutrient metabolism including glucose, fatty acid and AA [21,22]. Insulin-like growth factor 1 (IGF-1) and insulin-like growth factor 2 (IGF-2) are the major anabolic agents that contribute to tissues, especially muscle nutrient metabolism in teleost fishes [23,24,25].

Previous studies demonstrated that FM replacement had a profound effect on fish muscle nutrient deposition. Our study on turbot (*Scophthalmus maximus* L.) presented that FM replaced by soybean meal and meat and bone meal at 45% significantly decreased the muscle nutrients’ deposition by activating the AAR signaling pathway [26]. Furthermore, a study on the juvenile blunt snout bream (*Megalobrama amblycephala*) found that 5% and 7% cottonseed protein hydrolysate-replaced FM could decrease the muscle glucose, lipid and AA metabolism in muscle through inhibiting the TOR signaling pathway and activating the AMP-activated protein kinase/sirtuin-1 (AMPK/SIRT1) pathway [27]. Mixed plant proteins substituted 40% of FM in turbot and also significantly inhibited the TOR signaling pathway and protein synthesis in the muscle [28]. Moreover, total FM replaced by plant protein blends could suppress the muscle growth of Senegalese sole (*Solea senegalensis*) via altering the expression pattern of genes involved in the GH-IGF signaling pathway [29]. Therefore, the muscle nutritive metabolism response was considered as the crucial index to fully evaluate the optimal alternative ratio for protein sources.

Cottonseed meal (CSM) is known as a non-grain protein source that has cheaper prices, steady supply, relatively higher protein content and a well-balanced AA profile [30,31]. Currently, it has become a popular alternative protein source to replace FM in aquafeeds [32,33]. In the past several years, aquatic nutritionists have carried out research on the application of CSM in different aquafeeds [34,35]. All the studies demonstrated that CSM replacement proportions in feeds were quite different among different species. A study on black seabass (*Centropristis striata*) presented that CSM prepared by a glandless seed could successfully replace dietary 100% FM [36]. However, Bu et al. reported that CSM inclusion over 25.3% could significantly depress the growth performance in the Ussuri catfish *Pseudobagrusus suriensis* [37]. The studies mentioned above only focused on the effect of CSM replacement on growth performance and feed utilization. More attention should also be paid to the changes of muscle nutritive metabolism in order to accurately evaluate the replacement efficiency of CSM.

Golden pompano (*Trachinotus ovatus*) is widely cultivated in Guangdong, Fujian and Hainan provinces. Due to its advantage of growing fast, favorable taste, good nutrient profile and suitability for culture, it has become a commercially important marine fish species in China [38]. In the past decade (2010–2019), golden pompano production in China ranged from 0.75 million tons to 1.68 million tons and was worth 20 billion yuan [39]. Therefore, the industry of golden pompano culture has bright market prospects and huge economic benefits [40]. Previously, our team did some research on the CSM of golden pompano. The results present that, based on growth performance and feed utilization, CSM could substitute 20% FM without adverse effect [41]. As mentioned above, muscle deposition and nutrient metabolism were also the important indicators to evaluate the efficiency of CSM in replacing FM. Therefore, the present study was conducted to explore the physiobiochemical changes and underlying mechanisms of muscle nutrient metabolism after the FM was replaced by CSM. This study will provide a more comprehensive theoretical basis for the rational addition of CSM in the golden pompano diet.

## 2. Results

### 2.1. Proximate Composition of Muscle

The muscle proximate compositions of golden pompano after being fed with different diets were shown in Table 1. The values of crude protein (CP) and ash did not significantly differ among fish fed with four different experimental diets (*p* > 0.05). Meanwhile, compared with the control diet, the CSM20 and CSM40 diets also did not significantly change the moisture and crude fat (CF) of muscle (*p* > 0.05). Only the CSM60 diet increased the moisture and decreased CF (*p* < 0.05).

### 2.2. Free AAs Profile in Muscle

Changes of free AAs after fish were fed with different diets in muscle were presented in Table 2. Compared with that of the control diet, the concentration of leucine, threonine, lysine, histidine, arginine, glutamic acid, alanine, aspartic acid, serine and taurine were unaffected by three CSM-containing diets (*p* > 0.05). For other AAs, all the individual essential amino acids (EAAs) and non-essential amino acids (NEAAs) did not present significant differences among CSM0, CSM20 and CSM40 groups (*p* > 0.05), except phenylalanine, proline, tyrosine, NEAA and total amino acid (TAA) demonstrated significantly lower concentrations in CSM40 (*p* < 0.05). The CSM60 diet group presented a decreased free AA profile compared with the control group (*p* < 0.05).

### 2.3. Gene Expression Profile Related to Glucose Metabolism in Muscle

**Figure 1:**
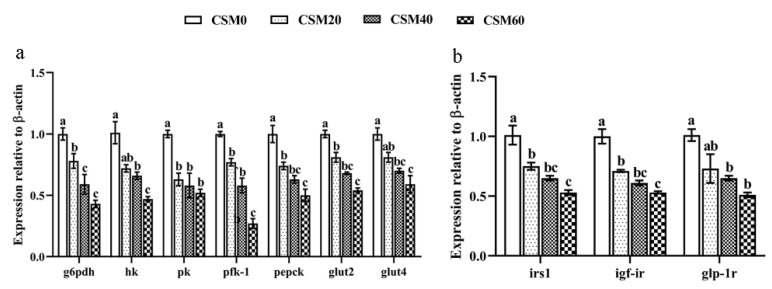
Diagrammatic representation of the gene expression pattern of (**a**) the key enzyme and transporters involved in glucose metabolism and (**b**) hormone receptors involved in insulin signaling pathway and glp-1r signaling in muscle after golden pompano (*Trachinotus ovatus*) were fed CSM0, CSM20, CSM40 and CSN60. Data were represented as mean ± SEM. Different letters above the bars denote significant differences between diet groups at the *p* < 0.05 level. Note: g6pdh, glucose-6-phosphate; hk, hexokinase; pk, pyruvate kinase; pfk-1, phosphofructokinase-1; pepck, phosphoenolpyruvate carboxykinase; glut2, glucose transport protein 2; glut4, glucose transport protein 4; irs1, insulin receptor substrate 1; igf-1r, insulin-like growth factor-1 receptor; glp-1r, glucagon-like peptide 1 receptor.

Figure 1 described the mRNA expression level of genes related to glucose metabolism in muscle after the golden pompano were fed diets containing different CSM levels. Compared with the CSM0 diet, the CSM20 diet had no effect on altering the gene expression of hexokinase (hk), glucose transport protein 4 (glut4) and glucagon like peptide 1 receptor (glp-1r) (*p* > 0.05). In contrast, the CSM20 diet significantly reduced the gene expression level of glucose-6-phosphate (g6pdh), pyruvate kinase (pk), phospho fructokinase-1 (pfk-1), phosphoenolpyruvate carboxykinase (pepck), glucose transport protein 2 (glut2), insulin receptor substrate 1 (irs1) and glucagon-like peptide 1 receptor (igf-ir) in comparison to the CSM0 diet (*p* < 0.05). Moreover, CSM40 and CSM60 diets also inhibited the glucose metabolism level more than the control diet (*p* < 0.05).

### 2.4. Effects on Muscle Lipid Metabolism by Experimental Diets

**Figure 2:**
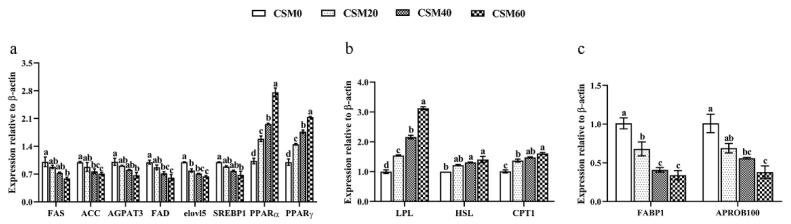
Effects of different experimental diets on the mRNA expression levels of (**a**) lipid anabolism, (**b**) lipid catabolism and (**c**) lipid transporters in muscle after the golden pompano (*Trachinotus ovatus*) were fed different diets. Data were represented as mean ± SEM. Different letters above the bars denote significant differences between diet groups at the *p* < 0.05 level. Note: FAS, fatty acid synthetase; ACC, acetyl-CoA carboxylase; AGPAT3, 1-acylglycerol-3-phosphate acyltransferase 3; FAD, fatty acyl desaturase; elovl5, elongase of very long-chain fatty acids 5; SREBP1, sterol regulatory element binding protein-1; PPARα, peroxisome proliferator activated receptors-alpha; PPARγ, peroxisome proliferator-activated receptors gamma; LPL, lipoprotein lipase; HSL, hormone-sensitive lipase; CPT1, carnitine palmitoyl transferase 1; FABP1, fatty acid binding protein 1; APROB100, apolipoprotein b 100.

Effects of different experimental diets on the mRNA expression levels of lipid anabolism are shown in Figure 2a. ANOVA did not reveal a significant influence of CSM20 on the expression of fatty acid synthetase (FAS), acetyl-CoA carboxylase (ACC), 1-acylglycerol-3-phosphate acyltransferase 3 (AGPAT3), fatty acyl desaturase (FAD) and sterol regulatory element binding protein-1 (SREBP1) in comparison to CSM0 diet (*p* > 0.05). Moreover, the CSM40 diet had no significant effects on altering the expression of FAS, AGPAT3 and SREBP1 (*p* > 0.05). However, compared with the CSM0 diet, three CSM containing diets significantly inhibited the mRNA expression of elovl5 and improved the peroxisome proliferator-activated receptors’ alpha (PPARα) and peroxisome proliferator-activated receptors’ gamma (PPARγ) expression (*p* < 0.05).

The muscle gene expression pattern of the key regulators involved in lipid catabolism after the fish were fed with different diets is presented in Figure 2b. Compared to the control diet, the mRNA expression level of hormone-sensitive lipase (HSL) was not significantly changed by the CSM20 group (*p* > 0.05). Meanwhile, the expression of lipoprotein lipase (LPL) and carnitine palmitoyl transferase 1 (CPT1) of the fish fed CSM20, CSM40 and CSM60 diets were more significantly up-regulated than the control diet (*p* < 0.05).

The effects of dietary CSM substitution on the transcriptional expression level of lipid transporters is illustrated in Figure 2c. No significant differences were detected of the mRNA expression level of apolipoprotein b 100 (APROB100) between the CSM0 and CSM20 groups (*p* > 0.05). However, compared with the CSM0 diet, other diets significantly inhibited the transcriptional expression level of the fatty acid binding protein 1 (FABP1) (*p* < 0.05).

### 2.5. Gene Expression of AA Transporter and Small Peptide Transporter

**Figure 3:**
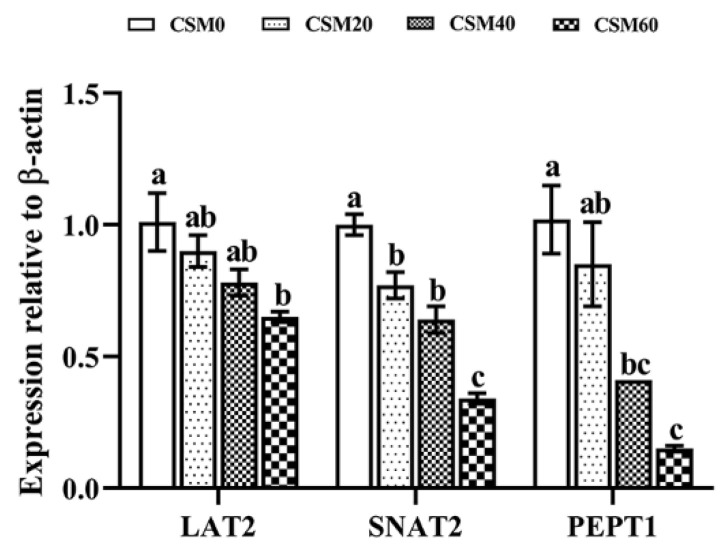
LAT2, SNAT2 and PEPT1 expression in muscle of juvenile golden pompano (*Trachinotus ovatus*) after fed test diets for 6 weeks. Data were represented as mean ± SEM. Different letters above the bars denote significant differences between diet groups at the *p* < 0.05 level. Note: LAT2, L-type amino acid transporter 2; SNAT2, sodium-coupled neutral amino acid transporter 2; PEPT1, oligopeptide transporter1.

The gene expression patterns of L-type amino acid transporter 2 (LAT2), sodium-coupled neutral amino acid transporter 2 (SNAT2) and oligopeptide transporter1 (PEPT1) had a decreasing trend with an increasing CSM replacement level (Figure 3). Compared with the CSM0 diet, the CSM20 diet had no effect on changing the gene expression of LAT2 and PEPT1 (*p* > 0.05), and CSM40 diet did not significantly down-regulate the expression of LAT2 (*p* > 0.05). However, fish fed the CSM20 diet demonstrated significantly lower levels of SNAT2 than the fish fed the CSM0 diet (*p* < 0.05).

### 2.6. The mRNA Expression of the Genes Related to GH-IGF Axis

**Figure 4:**
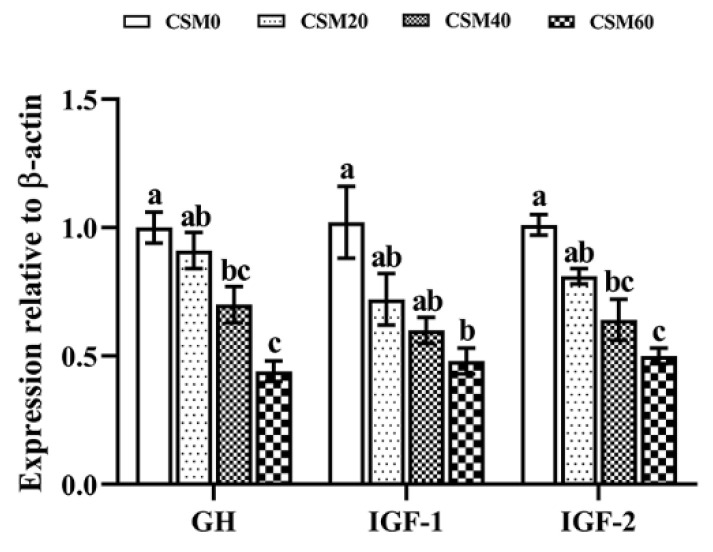
Different diets affected the relative gene expression of the key regulators of GH-IGF-1 axis in muscle of juvenile golden pompano (*Trachinotus ovatus*). Data were represented as mean ± SEM. Different letters above the bars denote significant differences between diet groups at the *p* < 0.05 level. Note: GH, growth hormone; IGF-1, insulin-like growth factor-1; IGF-2, insulin-like growth factor-2.

The transcription expression levels of key regulators of GH-IGF axis were significantly affected by dietary treatments (Figure 4). No significant differences were found between CSM0 and CSM20 groups in the growth hormone (GH) and IGF-2 (*p* > 0.05). Moreover, the expression of IGF-1 was not significantly down-regulated in fish fed the CSM20 diet and CSM40 diet compared to that of fish fed CSM0 (*p* > 0.05). The CSM60 diet caused a more suppressed GH, IGF-1 and IGF-2 expression pattern than the CSM0 diet (*p* < 0.05).

### 2.7. Regulations of the TOR and AAR Signaling Pathways

**Figure 5:**
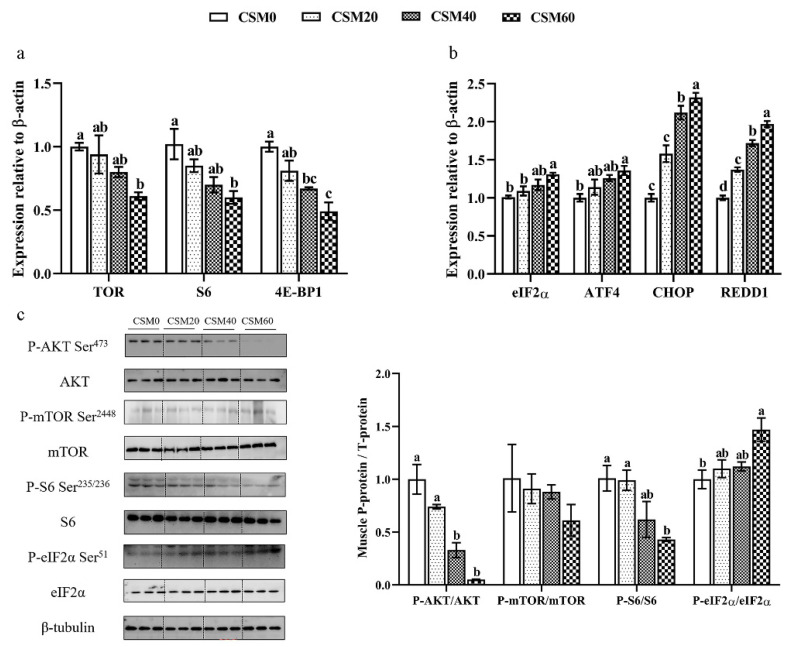
The mRNA expression and protein phosphorylation level of key regulators related to target of rapamycin (TOR) and amino acid response (AAR) signaling pathways in muscle. (**a**) mRNA expression level of TOR signaling pathway; (**b**) mRNA expression of AAR signaling pathway; (**c**) protein phosphorylation level of TOR and AAR signaling pathways. Data were represented as mean ± SEM. Different letters above the bars denote significant differences between diet groups at the *p* < 0.05 level. Note: TOR, target of rapamycin; S6, S6 Ribosomal Protein. 4E-BP1, eukaryotic initiation factor 4E-binding protein 1; eIF2α, initiation elongation factor alpha; ATF4, activating transcription factor 4; CHOP, channelopsin2; REDD1, regulated in development and DNA damage responses 1; AKT, protein kinase B.

Transcription and protein phosphorylation level of key regulators in TOR and AAR signaling pathways after the feeding trials were presented in Figure 5. In comparison to the control diet, relative mRNA expression of TOR, S6 Ribosomal Protein (S6) and eukaryotic initiation factor 4E-binding protein 1 (4E-BP1) were unaffected by the CSM20 diet (*p* > 0.05). Meanwhile, the CSM40 diet led to a significantly lower transcriptional level of 4E-BP1 (*p* < 0.05) but did not have marked effects on TOR and S6 expression (*p* > 0.05). For the gene expression of detected regulators in the AAR signaling pathway, no significant difference of initiation elongation factor alpha (eIF2α), activating transcription factor 4 (ATF4) and channelopsin2 (CHOP) mRNA expressions were found between fish fed CSM0 and CSM20 diets (*p* > 0.05). In addition, a significant difference of eIF2α and ATF4 mRNA expressions were detected between group CSM40 and group CSM0 (*p* > 0.05).

For the protein phosphorylation analysis, dietary inclusion of CSM did not affect protein phosphorylation level of mTOR after 6 weeks feeding (*p* > 0.05). Meanwhile, CSM20 diet did not had significant effects on altering the protein phosphorylation level of protein kinase B (AKT), S6 and eIF2α (*p* > 0.05) and CSM40 diet did not significantly change the expression pattern of S6 and eIF2α in comparison to CSM0 diet (*p* > 0.05). Meanwhile, the CSM60 diet markedly decreased the phosphorylation level of AKT, S6 and elevated the phosphorylation level of eIF2α (*p* < 0.05).

## 3. Discussion

The present study was part of a study exploring the optimum substitution ratio of CSM in golden pompano diets, and it used one growth trial as a research basis that determined the effects of CSM-replaced FM in growth performance and feed efficiency. In fish, the growth is dependent on muscle deposition, which largely related to the muscle nutritive metabolism [42,43]. Therefore, the current study was conducted to evaluate the effects of the CSM substituted FM on muscle nutritive metabolism as well as its potential mechanism.

### 3.1. CSM Substitution Alter Muscle Nutrient Composition and Free AAs Profile

Muscle is the main site of nutritive deposition for fish species. CP and CF are the important indicators that reflect the nutritive deposition after the fish are fed with different diets [44]. Our present study displayed that compared with the control diet, the CSM20 and CSM40 diets had no effect on changing the moisture, CP, CF and ash content of muscle. Only the fish fed with the CSM 60 diet had significantly increased the moisture and decreased CF of muscle, with the same results for the fermented soybean meal replacement in Japanese seabass (*Lateolabrax japonicus*) [45] and rapeseed meal in *Pseudobagrusus suriensis* [46]. Furthermore, it has been reported that the free AA profile is the key driver to regulate protein and other nutrient metabolisms in muscle [47]. Our current observations indicated that CSM20 and CSM40 diet did not markedly vary the concentration of most individual AA and EAA. Meanwhile, the CSM60 diet reduced the concentration of EAA, NEAA and TAA in muscle. These results were consistent with our previous studies on turbot and shrimp demonstrating the great variation on free AA after a higher percentage of FM was replaced by alternative proteins [48,49]. We envisaged that the changes of free AA profile after 60% FM replaced by CSM might be partially due to the unbalanced AA composition of higher CSM containing diets [50,51]. A lower level of free AA in muscle not only decreased the bricks for protein synthesis and antioxidative capacity but also disordered the glycolipid metabolism [52,53]. Moreover, the present study also demonstrated that the CSM 60 diet significantly decreased the glycine and tyrosine concentration more than the CSM0 diet. Glycline and glutamic acid play important physiological roles in glutathione (GSH) synthesis, which can protect fish against free radicals by increasing the activity of the antioxidant enzyme of fish [54]. Their results were consistent with our previous study that higher CSM replacement could affect fish immunity [41]. Moreover, data on muscle proximate composition also hinted at lipid deposition changes after fish fed with the CSM60 diet. Thus, we next investigate the changes of the glycolipid metabolism transcriptional level in muscle after golden pompano were fed with gradient CSM addition diets.

### 3.2. CSM Substitution-Modified Glycolipid Metabolism

It is well known that nutrient compositions are the most important indicators to estimate muscle growth and quality [55,56]. The inhibition of muscle nutrient deposition due to glycolipid metabolism disorder is also the bottleneck of FM replacement [57,58]. Data on the present study illustrated that the mRNA expression of key enzymes and transporters in glycolipid metabolism were extremely sensitive to the dietary protein sources. Only 20% of the CSM replacement could significantly reduce the enzymes’ expression of glucose metabolism. However, for lipid metabolism, less than 60% of the CSM replacement did not alter the mRNA expression levels of FAS, AGPAT3 and SREBP1. A similar observation could be found in the study of soy bean protein for cod *Gadus morhua* [59] and rapeseed meal diet for Chinese perch *Siniperca chuatsi* [60]. Furthermore, our recent study on largemouth bass (*Micropterus salmoides*) also presented that the glucose metabolism was more sensitive to feed compositions [61]. Similar to the largemouth bass, the golden pompano was also a carnivorous fish, which had dysglycemia after the higher FM was substituted by alternative proteins. CSM as a plant protein source has a higher content of carbohydrate compared with FM [62]. That might give a reasonable explanation for the dramatic changes of glucose metabolism after the golden pompano were fed with a higher CSM containing diet. Studies on mammal and fish species demonstrated that glycolipid metabolism could be modulated by nutrient sensing signaling pathways [63,64,65]. Therefore, in order to reveal the mechanisms of the changed nutrient metabolism, we next investigated the response mechanism of nutrient sensing signaling pathways after FM substituted by CSM.

### 3.3. CSM Substitution through Nutrient Sensing Signaling Pathways to Regulate Glycolipid Metabolism

AA transporters are known to regulate intracellular AA concentrations and TOR activity in lysosomes, which are regarded as the first sensors of dietary nutrients [66]. The present study displayed the variation in the AA profile of muscle after the fish were fed with different CSM containing diets. Therefore, we further measured the mRNA expression level of AA transporters in muscle tissue to explain the underlying mechanism of the changes in the AA profile. Data on the AA transporters’ expression demonstrated that CSM40 and CSM60 diets significantly decreased the mRNA expression of LAT2, SNAT2 and PEPT1. Similarly, in blunt snout bream [67] and turbot [68], FM replaced by alternative proteins also decreased the AA transporters’ expression. A lower level of AA transporters reduced the AA transmembrane transport efficiency, resulting in an overall decrease in muscle AA levels [69,70]. A restricted AA level in muscle would directly affect the activity of nutrient sensing signaling pathways, and further changed the body growth and metabolism [27,71]. Therefore, we next tried to explore the activity of nutrient sensing signaling pathways after the golden pompano were fed with different diets.

In mammals, it has been well revealed that TOR and AAR were two complementary signalings, which were responsible for sensing the nutrient level to regulate body protein synthesis and nutrient metabolism [72,73,74]. Moreover, GH and its downstream mediator, IGF-1, constructed a pleotropic axis through the TOR signaling pathway regulating growth, metabolism and organ function [75,76]. Recent studies from our laboratory reported that in largemouth bass, a dietary AA level could through TOR, AAR and GH-IGF signaling to regulate fish growth performance and glycolipid metabolism [53]. Likewise, the present study in golden pompano also demonstrated that dietary protein sources had profound effects on changing nutrient sensing signaling pathways. In detail, compared with the control diet, the CSM60 diet significantly decreased the mRNA expression level and protein phosphorylation level of the key regulators involved in GH-IGF and TOR signaling pathways and increased the mRNA expression level and protein phosphorylation level of the key regulators involved in the AAR signaling pathways in muscle. Our previous study focused on the growth performance after golden pompano fed with the CSM substitution diet also presented a similar changing pattern in the liver [41]. In Olive Flounder (*Paralichthys olivaceus*), the FM replacement modified the growth performance through the IGF system as well [77]. Additionally, the FM substituted with a composite mixture of shrimp hydrolysate and plant proteins in largemouth bass also presented that the appropriate FM replacement with composite mixture could improve the growth performance through activating the TOR signaling pathway [78]. The suppressed TOR signaling pathway would inhibit the whole-body total protein synthesis, especially in muscle. The reduced protein synthesis directly led to decreased muscle deposition. On the other hand, glycolipid metabolism was also inhibited through regulating TOR, AAR and GH-IGF pathways, which resulted in a decrease in the nutrients’ deposition of muscle. All the data confirmed our results, in which higher CSM supplementation disturbed nutrient sensing signaling and further inhibited muscle nutrient metabolism. This may explain why CSM could not successfully replace over 40% FM in the golden pompano diet.

## 4. Materials and Methods

### 4.1. Experimental Diets

Four isonitrogenous (42.5% crude protein) and isolipidic (14% crude lipid) diets were produced with CSM gradient-replaced FM. Dietary protein was supplied by FM, CSM, corn-gluten meal and poultry byproduct meal. Fish oil, soybean oil and soybean lecithin were used as lipid sources. The control diet (CSM0 diet) was based on FM, and other diets were named the CSM20 diet, CSM40 diet and CSM60 diet, in which CSM was used to replace 20%, 40% and 60% FM, respectively. All dry ingredients were grounded into powder with 320-µm and then mixed thoroughly. An appropriate amount of oil was added in order to meet the requirement of lipid for fish. The resulting doughs were extruded through a pelletizer (F-26, South China University of Technology, Guangzhou, China) after being further homogenized. All the diets were dried at 45 °C to an approximately 10% moisture level and stored in a refrigerator at −20 °C before the feeding trial. The proximate composition of all the experiment diets was given in Table 3. The essential amino acids’ composition of each experiment diet was given in Table 4.

### 4.2. Fish and Feeding Management

The experiment was performed with three hundred and sixty juvenile golden pompanos that were obtained from the Dayawan Fish Farm (Guangdong, China). Before the start of the experiment, all fish were fed with the CSM0 diet for two weeks to adapt to the experimental conditions. Before the experiment, 360 golden pompanos were fasted for 24 h and weighed. Fish with a mean initial weight of 28.42 ± 0.02 g were randomly distributed to 12 sea cages (1.5 m × 1.5 m × 1.5 m). Each experiment diet was randomly assigned to three replicates, and 30 golden pompanos were in each replicate. All treatments were fed their respective feeds twice daily (07:00 and 17:00) to apparent satiation for 6 weeks. The physiobiochemical properties of the water were recorded daily. The temperature and dissolved oxygen of water were maintained at 28–30 °C and 5.0–6.0 mg/L.

### 4.3. Sample Collection

At the end of the feeding trial, all of the golden pompano were fasted for 24 h and weighed. Four fish from each cage were rapidly anesthetized with eugenol (Shanghai Medical Co., Ltd., Shanghai, China), and the dorsal muscle of the fish was cut from the posterior of the anus to the posterior of the dorsal fin, then was quickly collected and immediately frozen. Samples of the muscle molecular analysis were kept in an RNAase tube (Axygen, Union City, CA, USA), snap-frozen in liquid nitrogen and then reversed at −80 °C until gene expression analysis.

### 4.4. AA Composition of Diet and Proximate Composition; Free AAs of Muscle

Diets were dried by a freezer dryer (ALPHA1-2 LD plus, Christ Co., Ltd., Berlin, Germany) in order to measure the AA compositions. The L-8900 AA analyzer (Hitachi, Tokyo, Japan) was used for analysis after all samples were digested with 6 M HCl for 22 h (Hitachi, Japan).

The proximate compositions of the muscle were determined, followed by the methods reported by Yang [49]. The moisture content was conducted by drying samples to a constant weight in an oven at 105 °C and calculating it as a percentage. The crude lipid was measured by a petroleum ether extraction (B.P. 30–60 °C, 3 h) in a SoxtecTM 2055 extraction. The crude protein was analyzed through a Dumas nitrogen determination apparatus (DT autosampler, Europe Gerhardt Company, Königswinter, Germany). The ash was determined by incinerating samples in a muffle furnace (FO610C, Yamato Scientific Co., Ltd., Tokyo, Japan) at 550 °C for 12 h until the samples were at a constant weight. Moreover, the free AA composition in the muscle was performed by an auto AA analyzer (LA8080; Hitachi, Tokyo, Japan). The muscle sample (0.2 g) and 10% hydrochloric acid were mixed completely and then ground for 5 min. After being centrifuged at 12,000× *g* at 4 °C for 15 min, the supernatants were filtered through 0.22-µm filters for the free AA concentration analysis.

### 4.5. RNA Extraction and cDNA Synthesis

Total RNA was isolated from the muscle sample using Trizol Reagent (Vazyme Biotech Co., Ltd., Nanjing, China) according to the manufacturer’s instructions. After being eluted in diethyl pyrocarbonate (DEPC)-treated water, the RNA samples were subjected to electrophoresis on 1.2% agarose gel to confirm the integrity, and RNA quantity and quality was assessed as a RNA concentration and 260/280 nm absorbance ratio by using a NanoDrop 2000 spectrophotometer (Thermo, NanoDrop Technologies, Wilmington, DE, USA). The cDNA was generated from 1 µg of total RNA by using a Prime Script RT reagent Kit (Vazyme Biotech Co., Ltd., China). Reaction conditions were recommended by the manufacturer’s introductions. The cDNA was then diluted with DEPC water to 100 ng/µL and used for real-time qPCR to determine the gene expression levels in muscle.

### 4.6. mRNA Expression Analysis (RT-qPCR)

The primers used in this study were given in Table 5. Real-time PCR assays were performed on a CFX96 real-time PCR equipment (CFX96, BIO-RAD, Berkeley, CA, USA) in a total volume of 20 µL containing 10 µL of Hiff^®^ qPCR SYBR Green Master Mix (Yeasen, Shanghai, China), 3 µL of diluted cDNA, 0.5 µL of each primer and 6 µL of DEPC water. The amplification reaction was initially conducted at 95 °C for 2 min, followed by 40 cycles of denaturation at 95 °C for 15 s and annealing at 58 °C for 30 s, and finally 72 °C for 20 s. The specificity was confirmed by the melt curve, and the relative mRNA expression levels of target genes were analyzed using the 2^−ΔΔCt^ method. β-actin was used as the internal reference gene to normalize the mRNA expression of genes, and the relative gene expression is relative to the control group.

### 4.7. Western Blotting

The Western blotting was performed according to Xu’s study [48]. The BCA protein assay kit (Beyotime, Biotechnology, Shanghai, China) was used to estimate protein concentrations of samples following the manufacturer’s guide. The relative protein expression of total AKT, phosphor-AKT, total TOR, phosphor-TOR, total S6, phosphor-S6, total eIF2α and phosphor- eIF2α were analyzed by sodium dodecyl sulfate-polyacrylamide (SDS-PAGE) and then transferred onto 0.45-µm polyvinylidene fluoride (PVDF) membranes (Millipore, St. Louis, MO, USA) by electrophoretic transfer for 1 h at 100 V. After that, the PVDF membranes were blocked with 5% non-fat milk in Tris-buffered saline in tween 20 (TBST) containing 20 mM Tris-HCl, 500 mM NaCl and 1% Tween-20 at room temperature for 1 h. The membranes were incubated in each primary antibody overnight at 4 °C. After washing the membranes three times with TBST, horseradish peroxidase-labeled secondary antibodies (1:10,000 dilution) was added and incubated for 1 h at room temperature. After washing again with TBST three times, the membranes were then treated with enhanced chemiluminescence (ECL) regents (Beyotime, Biotechnology, China) to visualize the protein bands. Protein expression was quantified with NIH Image 1.63 software, and β-tublin was used as reference.

### 4.8. Statistical Analysis

The statistical analysis was performed using the software SPSS 19.0 (IBM SPSS Statistics, Version 19.0, Armonk, NY, USA), and the analysis results were presented as mean ± standard error of mean (SEM). Prior to analysis, the homogeneity of the variance of measurement data was confirmed. If the data found did not comply with the parametric assumption of normality and homogeneity of variance, data transformations (such as logarithms, square roots and reciprocals) were applied to meet the ANOVA criteria. Significant differences among treatment were analyzed by one-way analysis of variance (ANOVA), followed by Tukey’s multiple range test. For all cases, the level of significance was determined as *p* < 0.05. The figures in this paper were made using GraphPad Prims 8.0 (GraphPad Software Inc., USA).

## 5. Conclusions

Over all, the present results suggested that CSM could substitute 20–40% FM without affecting the muscle nutritive deposition. All data supplemented powerful support for our previous conclusion that CSM could successfully replace 20% FM based on growth performance. These two studies presented a comprehensive evaluation of the optimal substitution ratio of CSM to replace FM in the golden pompano. Moreover, the underlying mechanism of over 40% FM replaced by CSM that reduced muscle nutrient deposition was also investigated in this study. Inhibited GH-IGF, TOR and activated AAR suppressed glycolipid metabolism and further decreased nutrient deposition in muscle tissue. However, more in-depth mechanisms should be further elucidated in future studies.

## Figures and Tables

**Table 1:** Muscle proximate composition of golden pompano fed diets containing various levels of CSM.

	Diet Groups (% Wet Weight)				*p*-Value
	CSM0	CSM20	CSM40	CSM60	
Moisture	69.34 ± 0.080 ^b^	69.47 ± 0.32 ^ab^	70.24 ± 0.26 ^ab^	70.51 ± 0.21 ^a^	0.018
CP	20.03 ± 0.17	20.72 ± 0.51	19.84 ± 0.88	20.51 ± 0.23	0.208
CF	8.91 ± 0.65 ^a^	7.21 ± 0.026 ^ab^	7.21 ± 0.57 ^ab^	6.83 ± 0.23 ^b^	0.044
Ash	1.26 ± 0.027	1.33 ± 0.026	1.25 ± 0.018	1.31 ± 0.058	0.407

The results were presented as mean ± SEM. Means in the same row with different superscript letters are significantly different (*p* < 0.05). Note: CP, crude protein; CF, crude fat.

**Table 2:** Free amino acid profile in muscle of golden pompano fed different experimental diets.

	Diet Groups (ng/mg)				*p*-Value
	CSM0	CSM20	CSM40	CSM60	
Met	43.71 ± 0.93 ^a^	44.84 ± 1.13 ^ab^	40.89 ± 1.33 ^ab^	33.22 ± 1.96 ^b^	0.001
Phe	43.62 ± 3.19 ^a^	33.38 ± 1.77 ^ab^	31.61 ± 0.37 ^b^	30.93 ± 2.96 ^b^	0.017
Val	167.53 ± 10.73 ^a^	139.53 ± 11.59 ^ab^	141.54 ± 2.90 ^ab^	112.07 ± 10.51 ^b^	0.023
Ile	127.65 ± 10.40 ^a^	106.72 ± 7.35 ^ab^	104.23 ± 1.03 ^ab^	83.89 ± 7.86 ^b^	0.022
Leu	205.13 ± 18.18	172.39 ± 12.98	166.00 ± 1.41	148.16 ± 13.30	0.07
Thr	178.37 ± 6.70	148.73 ± 16.26	141.79 ± 2.33	137.10 ± 8.44	0.067
Lys	319.69 ± 20.04	368.12 ± 8.13	335.26 ± 27.18	309.99 ± 3.01	0.174
His	63.99 ± 1.81 ^ab^	67.40 ± 1.08 ^a^	58.14 ± 5.29 ^ab^	50.96 ± 2.85 ^b^	0.029
Arg	62.77 ± 2.20	66.22 ± 0.27	60.94 ± 5.15	62.51 ± 1.96	0.655
EAA	1212.48 ± 40.76 ^a^	1147.33 ± 58.05 ^ab^	1080.40 ± 31.39 ^ab^	968.84 ± 38.64 ^b^	0.021
Glu	400.00 ± 38.53	405.77 ± 30.42	413.25 ± 20.72	446.68 ± 7.68	0.632
Gly	1866.44 ± 86.41 ^a^	1854.01 ± 18.75 ^a^	1564.49 ± 46.94 ^ab^	1349.20 ± 119.88 ^b^	0.004
Pro	96.91 ± 7.27 ^a^	81.52 ± 0.47 ^ab^	76.64 ± 0.59 ^b^	71.41 ± 3.19 ^b^	0.01
Ala	449.77 ± 11.61	439.88 ± 2.36	429.38 ± 17.31	461.63 ± 4.09	0.251
Asp	15.11 ± 0.84	16.11 ± 1.04	12.72 ± 0.80	12.36 ± 0.86	0.046
Tyr	38.81 ± 2.47 ^a^	29.87 ± 0.98 ^ab^	29.04 ± 0.12 ^b^	28.90 ± 2.80 ^b^	0.021
Ser	187.81 ± 3.59	180.68 ± 5.42	177.38 ± 10.78	174.06 ± 8.75	0.64
Tau	1738.72 ± 59.66	1762.94 ± 46.02	1776.66 ± 8.59	1550.36 ± 85.14	0.072
NEAA	4793.57 ± 62.88 ^a^	4771.29 ± 67.07 ^a^	4479.57 ± 27.84 ^b^	4094.60 ± 46.06 ^b^	0
Total AA	6006.05 ± 93.11 ^a^	5918.42 ± 80.25 ^a^	5559.97 ± 24.53 ^b^	5063.44 ± 74.82 ^c^	0

The results were presented as mean ± SEM. Means in the same row with different superscript letters are significantly different (*p* < 0.05). Note: Met, methionine; Phe, phenylalanine; Val, valine; Ile, isoleucine; Leu, leucine; Thr, threonine; Lys, lysine; His, histidine; Arg, arginine; EAA, essential amino acids; Glu, glutamine; Gly, glycine; Pro, proline; Ala, alanine; Asp, asparagine; Tyr, tyrosine; Ser, serine; Tau, taurine; NEAA, non-essential amino acids; TAA, total amino acids.

**Table 3:** Experimental diet formulations.

*Ingredients*	Diets (% Dry Weight)			
	CSM0	CSM20	CSM40	CSM60
Fishmeal	25.00	20.00	15.00	10.00
Cottonseed meal (CSM)	0.00	5.00	10.00	15.00
Corn gluten meal	13.00	13.00	13.00	13.00
Poultry by-product meal	11.00	11.00	11.00	11.00
Soybean meal	8.50	8.50	8.50	8.50
Peanut meal	6.50	6.50	6.50	6.50
Wheat meal	17.50	17.50	17.50	17.50
Fish oil	1.50	2.00	2.40	2.80
Soybean oil	5.00	5.00	5.00	5.00
Soybean lecithin	2.50	2.50	2.50	2.50
Monocalcium phosphate	1.50	1.70	1.90	2.10
Lysine	0.28	0.45	0.60	0.75
Methionine	0.10	0.15	0.20	0.25
Threonine	0.01	0.03	0.05	0.07
Squid paste	1.50	1.50	1.50	1.50
Mineral premix ^1^	1.50	1.50	1.50	1.50
Vitamin premix ^2^	0.50	0.50	0.50	0.50
Chromium trioxide	0.10	0.10	0.10	0.10
Lutein	0.10	0.10	0.10	0.10
Antioxidant	0.05	0.05	0.05	0.05
Mold inhibitor	0.10	0.10	0.10	0.10
Cellulose	3.76	2.82	2.00	1.18
***Proximate composition***				
DM (%)	90.24	89.98	89.91	90.12
Crude protein (%)	42.42	42.51	42.58	42.66
Crude lipid (%)	14.00	14.09	14.07	14.05

^1^ Mineral premix (mg/kg diet): NaF, 2 mg; KI, 0.8 mg; CoCl_2_·6H_2_O (10 g/kg), 50 mg; CuSO_4_·5H_2_O, 10 mg; FeSO_4_·H_2_O, 80 mg; ZnSO_4_·H_2_O, 50 mg; MnSO_4_·H_2_O, 60 mg; MgSO_4_.7H_2_O, 1200 mg; Ca(H_2_PO_4_)_2_·H_2_O, 3000 mg; NaCl, 100 mg; zeolite, 15,447 mg. ^2^ Vitamin premix (mg/kg diet): thiamin, 25 mg; riboflavin, 45 mg; pyridoxine HCl, 20 mg; vitamin B12, 0.1 mg; vitamin K3, 10 mg; inositol, 800 mg; pantothenic acid, 60 mg; niacin acid, 200 mg; folic acid, 20 mg; biotin, 1.20 mg; retinal acetate, 32 mg; cholecalciferol, 5 mg; α-to-copherol, 120 mg; ascorbic acid, 2000 mg; choline chloride, 2500 mg; ethoxyquin 150 mg; wheat middling, 14,012 mg.

**Table 4:** The essential amino acid composition of the experimental diets.

*Amino Acids*	Diets (% Dry Weight)			
	CSM0	CSM20	CSM40	CSM60
Lys	1.90	1.92	1.92	1.91
Met	0.70	0.70	0.70	0.70
Thr	0.88	0.88	0.88	0.88
Arg	1.29	1.51	1.73	1.95
His	0.47	0.49	0.51	0.53
Ile	0.84	0.81	0.79	0.77
Leu	1.51	1.48	1.45	1.42
Phe	0.89	0.94	0.99	1.04
Val	1.02	1.01	0.99	0.97
Cys	0.15	0.18	0.20	0.23
Tyr	0.66	0.65	0.65	0.64

**Table 5:** Primers used for determining gene expression.

Target Gene	Forward Sequence (5′–3′)	Reverse Sequence (5′–3′)
g6pdh	CTGTGGCAAAAGTTGGTGTG	CCTGATGATGTGAGGGATGA
hk	CCTTCCTCGTCTTTGTCATTT	TGTCCGTCTCATCCTGGTG
pk	TTTGCCAGTTTCATCCGCT	CCATCACGCCATCGCTCT
pfk-1	TGGGTGGGACCGTGATT	AGGTTGGTGATGCCTTTCTT
pepck	TGGAGTGTTTGTTGGAGCAG	CGAAGTTGTAGCCGAAGAAG
glut2	TCCTGTTTGCTGTGCTGCTT	GTTTTCCGTCCCTTGCG
glut4	AATGGCTGTGGCTGGCTT	AGGTTTTTCCCCGTGTTTCT
irs1	GCTCCACCCCTCTATTATCTCCT	GTACCTCCCACAGTTCCTCAGTC
igf-i r	TTCTGCTGTGCTCTTGTCT	GATGTTTTTGGTGTGGCT
glp-1 r	GGCAATCTCTCCTGTTCCC	AGCCTCTGCTTTTATTCGTG
FAS	GATGGATACAAAGAGCAAGG	GTGGAGCCGATAAGAAGA
ACC	GTTGTCAATCCCAGCCGATC	ATCCACAATGTAGGCCCCAA
AGPAT3	CTTCCTGTTTTGGGCCACTC	GTCGCCATAACTTGAGCCTG
FAD	GAACAATCCCACTTCAACG	AGGAATCCCATACTTCTCACA
elovl5	TACATGGTCACGCTCATTATCC	CCGTTCTGATGCTCCTTCTTTA
SREBP1	GAGCCAAGACAGAGGAGTGT	GTCCTCTTGTCTCCCAGCTT
PPARα	AATCTCAGCGTGTCGTCTT	GGAAATGCTTCGGATACTTG
PPARγ	TCAGGGTTTCACTATGGCGT	CTGGAAGCGACAGTATTGGC
LPL	TTTGTCCTTCCTCGTCACCA	AAGACAGCATCCTCTCCACC
HSL	TCATACCTCCACACCAACCC	GTCTCGCAGTTTCTTGGCAA
CPT1	CTTTAGCCAAGCCCTTCATC	CACGGTTACCTGTTCCCTCT
FABP1	CCAAGGACATCAAGCCAATTAC	TGGTGATTTCAGCCTCCTTAC
APROB100	AAAAGCCACAAGACGAAAGCA	GAAGCAGCAAAAGGCAGAGC
LAT2	CTCCCAGCAGCTTCTCACCAAAC	CTCGTGCCATCTTCATCTCCATC
SNAT2	CTGCTGGCTGCCCTTTTCGGATA	AGGACAGGTGCTGGTTGATGGAG
PEPT1	AACTGGTCTCCTCCAAACGC	GTTGGAGCCATTCCCACTGT
GH	CGGAGCAGTCAGAGTCTTCTACCT	TTCCACAGTAAAACAGTCATCATCAT
IGF-1	CGCAATGGAACAAAGTCGG	AGGAGATACAGCACATCGCACT
IGF-2	GCAAAGACACGGACCCCACT	CGAGGCCATTTCCACAACG
TOR	GGGTCTTATGAGCCAGTGCCAGG	CTTCAGGGTTGTCAGCGGATTGT
S6	GCACTGTCCCTCGCCGTCTT	CTGGGCTTCTTGCCTTCTTT
4E-BP1	ACACCCCAGCAGGAACTTT	GTGACCATCAACGACGCAG
eIF2α	TGTATTCCAGCACCTCAGCC	CGTGGTCGTCATCCGAGTAGA
ATF4	CTGCGTCACCCCTCAACTCC	CATTCGCTCCATCCACAACC
CHOP	CGGAGTTTCTGGATGTTTTGGA	AGGAGGAGGAAGAGGAGGATGA
REDD1	AGCCAAAGACTCAGAATGCG	TGAAAGGTGGGGACAAGGTA
β-actin	TACGAGCTGCCTGACGGACA	GGCTGTGATCTCCTTCTGC

## Data Availability

The data presented in this study are available within the article.
